# Selenium, selenoprotein P, and oxidative stress levels in SARS-CoV-2 patients during illness and recovery

**DOI:** 10.1007/s10787-022-00925-z

**Published:** 2022-02-14

**Authors:** A. Skesters, D. Kustovs, A. Lece, E. Moreino, E. Petrosina, K. D. Rainsford

**Affiliations:** 1 Scientific Laboratory of Biochemistry, Riga Stradiņš University, Riga, Latvia; 2 Laboratory of Statistic, Riga Stradiņš University, Riga, Latvia; 3 Biomedical Sciences, Biomedical Research Centre, Sheffield Hallam University, Sheffield, UK

**Keywords:** COVID-19, Oxidative stress, Free radicals, Selenium, Selenoprotein P

## Abstract

**Background:**

This study aimed to assess tendency of oxidative stress in COVID-19 patients depending on severity.

**Methods:**

The study was conducted with 80 post-COVID-19 disease patients and 40 acutely ill patients. Content of selenium in blood plasma was detected by a fluorimetric method with di-amino-naphthalene using acidic hydrolysis. Selenoprotein P, malondialdehyde and 4-hydroxynonenal and their metabolite adducts were evaluated by spectrophotometric methods using commercial assay kits.

**Results:**

Obtained results showed that selenium content in blood for post-COVID-19 disease patients was of a similar lower norm for Latvian inhabitants. Selenium and seleno-protein P contents for acute patients were significantly decreased compared with post-COVID-19 disease patients.

**Conclusion:**

In conclusion, COVID-19 involves induction of antioxidant systems—in case of severe disease, patients have significantly low concentration of selenium, seleno-protein P and higher level of oxidative stress, which, in turn, confirms the more intense formation of free radicals in the body.

## Introduction

SARS-CoV-2 is responsible for the COVID-19 pandemic that started in Wuhan, China, and has already claimed more than 4000 lives in Latvia (inhabitants in Latvia on 01.01.2019: 1.908.000, number of COVID-19 cases: 291.000 on 10.01.2022).

SARS-CoV-2 is a single-stranded RNA virus, such as Ebola virus, HIV, coxsackievirus, influenza, SARS, MERS (Zhang et al. 2020). Recent investigations, mostly conducted in the country of SARS-CoV-2 origin, China, showed an interaction between selenium (Se) level in the body and the SARS-CoV-2 virus. It has been shown that Se deficiency promotes mutations, replications and virulence of different RNA viruses (Moghaddam et al. 2020).

Despite the existing evidence, discussions are still taking place on what role, if any, Se may play in reducing the severity and mortality of COVID-19 infection. Studies of recent months show a close relationship between Se, Selenoprotein P (Sepp1) deficiency, oxidative stress level and COVID-19 disease incidence, severity and prognosis (Cheng and Prabhu 2019; Moghaddam et al. 2020; Saito 2020; Zhang et al. 2020). Se deficiency is thought to suppress the non-specific cell-mediated immune response and adaptive antibody response, which leads to dysregulation of the balanced host response. This increases the susceptibility to infections, with increased morbidity and mortality (Jayawardena et al. 2020). Se deficiency can therefore weaken the immune response to viruses and increase the virulence of particular viruses, also including coronaviruses. Meantime, viral infections correlate with increased reactive oxygen species (ROS) production. Oxidative stress can in fact be considered the hallmark of viral infections. ROS, on the other hand, can enhance viral replication (Saito 2020).

Se is fundamentally important to human health because it acts as an essential part of several major metabolic pathways, such as antioxidant defense functions, immune functions and is incorporated in more than 20 seleno-proteins (SelP). Sepp1 is one of the most important seleno-proteins. It is well known that Se has a protective effect against some forms of cancer, it decreases cardiovascular disease mortality, regulates the inflammatory mediators in pulmonary diseases, etc.

Besides, Se plays complex roles in the immune system, especially in the oxidative stress processes. The main pathways of ROS-producing systems include mitochondrial oxidative phosphorylation, phagocytic cell NADPH oxidase, xanthine oxidase. Se is an essential ultra-trace element, which is incorporated into more than 20 SelP, as seleno-cysteine in their active center (Guillin et al. 2019).

Sepp1 is an extracellular, monomeric glycoprotein that contains up to 10 seleno-cysteine residues in the polypeptide chain. In human plasma, it accounts for at least 40% of the total Se concentration. Sepp1 can bind to heparin, cell membranes and is associated with endothelial cells. Sepp1 is considered to function as an extracellular oxidant defense; in human plasma, it protects against peroxy-nitrite-mediated oxidation and reduces phospholipid hydro-peroxide in vitro. Its concentration in plasma varies much with Se intake as immunochemical assays have demonstrated, but other factors may also have an influence (Åkesson et al. 1994).

Some of these SelP are important for defense from viral infections, oxidative stress and participate in thyroid hormone metabolism. A recent investigation showed that Se deficiency is associated with greater susceptibility to viral RNA infections and more severe outcomes (Hiffler and Rakotoambinina 2020). Similar to Se, SelP such as Sepp1, are essential for an effective immune response to different infections.

Among the 25 SelP genes identified to date, several have important cellular functions in antioxidant defense, cell signaling and redox homeostasis. There are many ways in which the host metabolism could be affected during viral infections, leading to a dysregulation of redox homeostasis and therefore oxidative stress. Viral pathogens usually induce oxidative stress by increasing the generation of ROS and alternating the cellular ROS scavenging or dis-mutation systems. It is known that Se and SelP, however, play important roles in controlling redox homeostasis (Guillin et al. 2019).

Oxidative stress (OS) is a biological circumstance driven by the imbalance between pro-oxidant and antioxidant equilibrium (Taso et al. 2019). OS is caused by free radicals (FR), reactive oxygen species (ROS) and RNS which damage lipids, proteins, bio-membranes, DNA and many other macromolecules. The degree of lipid peroxidation is often used as an indicator of OS-mediated damage. Whereas, the concentration of malondialdehyde (MDA) and 4-hydroxynonenal (4-HNE) and their metabolite adducts in the whole blood, blood plasma, serum, other body liquors, and tissues are generally used as biomarkers of lipid peroxidation.

In recent decades, Latvian agriculture has mainly used mineral fertilizers produced in Finland and Sweden, which are enriched with microelements, including Se. This has increased the amount of trace elements, including Se, in local foods. Consequently, Se deficiency in the population of Latvia has decreased in the last 10−15 years (Kumerova et al. 1998, 2000).

Consequently, in the field of public health, one of the priority areas is the reduction of Se deficiency.

The aim of this study was to detect levels of Se, Sepp1, MDA and 4-HNE adducts in the acute period. First, in patients hospitalized in Pauls Stradiņš Clinical University Hospital in the COVID-19 department or intensive care unit, and patients 2 months following their discharge from the hospital. Thus, several hypotheses would be tested, including Se deficiency may reduce immunity and thus contribute to the high incidence of COVID-19; the degree of Se and/or Sepp1 deficiency may be directly related to the severity and prognosis of the disease; oxidative stress directly affects the incidence and severity of the disease.

## Materials and methods

The study was conducted with 80 post-COVID-19 disease patients and 40 acutely ill patients. The study was performed in accordance with The Declaration of Helsinki, Guidance on Good Clinical Practices, and applicable regulatory requirements. This clinical trial was part of the research Programme of Latvia “Clinical, biochemical, immunogenetic paradigms of COVID-19 infection and their correlation with socio-demographic, etiological, pathogenetic, diagnostic, therapeutically and prognostically important factors to be included in guidelines”. Content of Se in blood plasma was detected by a fluorimetric method with di-amino-naphthalene using acidic hydrolysis. Inter-assay and intra-assay coefficients of variation were < 12, as determined with a commercial TraceCERT Se Standard for AAS, cat No 89498-250ML, Sigma-Aldrich, USA and human reference serum sample Trace Elements Serum L—1, SeroNorm™, SERO AS, Billingstad, Norway. Other chemicals produced by Sigma-Aldrich, USA. SelP were detected using Human Sepp1 ELISA kit, Cat No CSBEL 021018 HU, CUSABIO BIOTECH CO, Ltd, Wuhan, China, according to the manufacturer’s instructions. MDA was detected using OxiSelect™ TBARS (MDA Quantitation) Assay kit, Cat No STA-330, Cell Biolabs, Inc, San Diego, CA, USA, to the manufacturer’s instruction. 4-HNE Adducts were detected using OxiSelect™ HNE Adduct Competitive ELISA kit, Cat No STA-838, Cell Biolabs, Inc, San Diego, CA, USA, according to the manufacturer’s instructions. The absorption spectrum was measured using multimodal microplate reader SPARK, TECAN, Austria. Blood samples for analysis were collected in BD Vacutainer LH 170 I.U Plus Blood Collection tubes, centrifuged at 1500 g + 4 ℃ for 10 min. Blood plasma was aliquoted into cryogenic tubes and frozen at − 80 ℃ prior to analysis.

## Results

A total of 120 patients qualified for analysis and were enrolled in this study. Blood plasma Se, Sepp1, MDA and 4-HNE adduct status were evaluated from all patients samples. Patients were divided into three groups: infected with COVID-19 in the spring–early summer period (1st wave), summer–autumn patients (2nd wave), and acutely ill patients, with two subgroups: patients treated in the COVID-19 unit and patients who were treated in the intensive care unit (20 + 20). Mean concentrations in the population of Se, Sepp1, MDA and 4-HNE adducts were obtained from previous years’ studies in practically healthy adults (*n* = 195, mean age 49, range 21–59) (Kumerova et al. 2000; Skesters and Voicehovska 2004). The results of the study showed statistically significant differences between both acute patients and those who were already ill. Results differed in both plasma Se, Sepp1 as well as HNE adducts as an indicator of oxidative stress. Compared to the accepted norm in practically healthy people, the content of Se and SelP was significantly lower in all groups, but the oxidative stress markers MDA and HNE were many times higher than the accepted upper limit.

Our results showed that Se content in blood for post-COVID-19 disease patients was of a similar lower norm for Latvian inhabitants with little differences between spring–summer (1st wave) and summer–autumn (2nd wave). Conversely, Se content for acute patients was significantly decreased compared with post-COVID-19 disease patients: 69.7 µg/L and 84.6 µg/L and 88.2 µg/L, respectively. Even greater changes are seen when patients treated in intensive care units are excluded. The plasma of these patients contained only 59.3 µg/L of Se.

Our study showed that the contents of Sepp1 in plasma were accordingly 4.5 mg/mL for acute patients, 5.5 mg/mL 1st wave and 6.8 mg/mL 2nd wave post-COVID-19 patients. New research reveals the crucial role of SePP1 in neuroprotection under oxidative stress.

Our results showed extremely high levels in all patients groups—acutely ill and post-COVID-19 groups. In all patient groups, plasma levels of MDA were many times higher than those of healthy people, 26.6 µM/L, 31.0 µM/L, and 26.6 µM/L accordingly.

A different picture is formed by determining the plasma concentration of 4-HNE adducts. 4-hydroxynonenal is a lipid peroxidation product derived from oxidized ω-6 polyunsaturated fatty acids such as arachidonic acid. 4-HNE is widely used as a marker of lipid peroxidation. The highest level is reached in the blood of acute patients, the concentration of 4-HNE is slightly different in the group of summer-autumn patients and even lower—in the group of spring–summer patients, respectively, 5.1 µg/L, 3.4 µg/L and 3.9 µg/L (Table 1).

**Table 1:** Results of investigated parameters in different groups

Parameter	Acute, *N* = 40^1^	Spring–summer wave, *N* = 40^1^	Summer–autumn wave, *N* = 40^1^	*p* value
Se	69.7 (20.8)^a^	84.6 (20.7)^b^	88.2 (27.2)^b^	0.001
Sepp1	4.5 (2.4)^b^	5.5 (2.2)^b^	6.8 (2.3)^a^	< 0.001
MDA	26.6 (10.8)	31.0 (18.6)	26.6 (10.8)	0.268
4-HNE Add	5.1 (2.4)^a^	3.4 (1.9)^b^	3.9 (1.8)^b^	< 0.001
Log(4-HNE Add)	1.5 (0.5)^a^	1.1 (0.5)^b^	1.3 (0.4)	< 0.001

All the parameters and results are available without bold

^1^Average (SD)

^2^ANOVA

## Discussion

The results obtained in the study regarding the concentration of Se and Sepp1 in the blood plasma can be assessed as low (diseased) and as extremely low—in acute patients. It is very important to detect this Se and Sepp1 deficiency in the body, especially in patients in intensive care, where the Se concentration in the body turned out to be catastrophically low 59.3 ± 16.1 µg*/*L, respectively, the extreme limits (min/max) were from 75.4 µg*/*L to 43.2 µg*/*L. A similar situation is observed for changes in Sepp1 levels during the course of the disease—the smallest change is in acute patients, slightly higher in diseased patients. This, in turn, indicates complications not only because of Se as an antioxidant and SelP as a transport protein, but also due to an overall antioxidant deficiency in life processes in the body. There can be insufficient synthesis of certain enzymes (GPx family), hormones and related promoters (5-deiodinase, thyroid hormones), other SelP, and also a major influence on the immune function and immune response to COVID-19 disease.

Jayanta Talukdar et al., already showed that natural antioxidants, such as astaxanthin (ASH), can work as therapeutic agents against inflammatory cytokine storm and reduce associated risks in COVID-19 infection (Talukdar et al. 2020). A synergistic therapeutic approach with the inclusion of natural immuno-suppressants to relieve the cytokine storm could offer a great treatment potential (Peter et al. 2021). Accordingly, published data on Se and SelP role in hyper-cytokinemia support the hypothesis that Se adequacy prevents excessive cytokine activation in infectious, inflammatory and oncological models (Tseng et al. 2013; Fehr et al., 2017; Qian et al., 2019). Moreover, for COVID-19 disease, an association between more-than-adequate selenium intake/status and a higher cure rate has been identified (Zhang et al. 2020). It is known that the acute infection phase in COVID-19 lasts only a few weeks in typical cases, which is comparable to the time frame over which daily doses of 1 mg Se have been used in sepsis and critical care applications (Hardy et al. 2012; Manzanares et al. 2013). Based on that, over a similar time period, a comparable supra-nutritional dose of selenium would be very unlikely to result in toxicity in COVID-19 patients and might be beneficial in those with moderate-to-severe symptoms. However, the potential benefit of such a strategy would need to be tested clinically, preferably in a randomized, controlled trial (Zhang et al. 2020).

## Conclusion

Based on the obtained results, we can conclude the following: in case of severe disease, the patient has a lower concentration of Se and Sepp1; the opposite tendency is observed in the case of oxidative stress—the more severe the course of the disease, the higher the level of oxidative stress, which, in turn, confirms the more intense formation of free radicals in the body.

After developing COVID-19, we also found decreased levels of Se and Sepp1 in the body after a period of two to three months, while high levels of oxidative stress (chronic) remained.

We agree with J. Zhang et al. on the need to use organic Se preparations and natural antioxidants as adjunctive therapy for preventive purposes, during illness and recovery (Zhang et al. 2020).
